# Combining an Electrochemical Continuous Glucose Sensor With an Insulin Delivery Cannula: A Feasibility Study

**DOI:** 10.1177/19322968241236771

**Published:** 2024-03-16

**Authors:** Cheng Yi Yuan, Bella Halim, Yee W. Kong, Jean Lu, Ralph Dutt-Ballerstadt, Peter Eckenberg, Ken Hillen, Anh Koski, Vlad Milenkowic, Emma Netzer, Varuni Obeyesekere, Solomon Reid, Catriona Sims, Sara Vogrin, Huan-Ping Wu, Thomas Seidl, David N. O’Neal

**Affiliations:** 1 Department of Medicine, St Vincent’s Hospital Melbourne, The University of Melbourne, Fitzroy, VIC, USA; 2 Pacific Diabetes Technologies, Portland, OR, USA

**Keywords:** insulin cannula, continuous glucose monitoring, continuous glucose monitor-insulin set, feasibility study

## Abstract

**Background:**

Combining a continuous glucose monitor with an insulin delivery cannula (CGM-IS) could benefit clinical outcomes. We evaluated the feasibility of a single-needle insertion electrochemical investigational CGM-IS (Pacific Diabetes Technologies, Portland, Oregon) in type 1 diabetes adults.

**Methods:**

Following 48 hours run-in using a Medtronic 780G in manual mode with a commercial insulin set, 12 participants commenced insulin delivery using the CGM-IS. A standardized test meal was eaten on the mornings of days 1 and 4. Venous samples were collected every 10 minutes one hour prior to and 15 minutes post-meal for four hours. CGM-IS glucose measurements were post-processed with a single capillary blood calibration during warm-up and benchmarked against YSI. A Dexcom G6 sensor was worn post-consent to study end.

**Results:**

Mean absolute relative difference (MARD) for the CGM-IS glucose measurements was 9.2% (484 paired data points). Consensus error grid revealed 88.6% within zone A and 100% in A + B. Mean (SD) % bias was −3.5 (11.7) %. There were 35 paired YSI readings <100 mg/dL cutoff and 449 ≥100 mg/dL with 81.4% within ±15 mg/dL or ±15%, and 89.9% within ±20 mg/dL or ±20%. Two cannula occlusions required discontinuation of insulin delivery: one at 70 hours post insertion and another during the day 4 meal test. Mean (SD) Dexcom glucose measurements during run-in and between meal tests was respectively 161.3 ± 27.3 mg/dL versus 158.0 ± 25.6 mg/dL; *P* = .39 and corresponding mean total daily insulin delivered by the pump was 58.0 ± 25.4 Units versus 57.1 ± 28.8 Units; *P* = .47.

**Conclusions:**

Insulin delivery and glucose sensing with the investigational CGM-IS was feasible. Longer duration studies are needed.

## Introduction

The management of those with type 1 diabetes mellitus (T1D) and many of those with type 2 diabetes mellitus (T2D) requiring insulin^1^ has been transformed by automated insulin delivery (AID) systems.^2,3^ However, glucose sensing and insulin delivery for all current AID systems employ separate insertion procedures. This imposes a physical burden on the user and may affect discontinuation rates. Combining glucose sensing and insulin delivery functions may address this burden, improving adherence with AID leading to better clinical outcomes.^4^

A recent review^5^ identified three potential factors compromising interstitial glucose measurements in proximity to insulin delivery: a high local insulin concentration increasing glucose uptake by adipocytes in the vicinity of the sensor; dilution of glucose levels by infused insulin; and insulin excipients interfering with sensor function.

Microdialysis and microperfusion catheters may address these barriers, but implementation in a clinical setting remains a challenge because of expense and complexity.^6,7^ Co-located but separate sensor and insulin cannula needle insertions with a separation distance sufficient to ensure that the sensor would be outside any potential insulin interference is feasible and safe,^8,9^ but the need for two penetrations, differing durability of the sensor and insulin cannula, and the size of the device impact utility.

More recently, Ward et al^10^ combined an electrochemical sensor and insulin delivery with a single-needle insertion. Insulin was infused into a minipig subcutaneously proximal to the sensing element minimizing its impact on glucose measurements. There was no evidence of a dilutional effect or a local hypoglycemic effect. Artifacts due to phenolic preservatives were observed but subsequently eliminated by modifying sensor chemistry to employ lower voltage potentials. Short duration studies under closely supervised conditions confirmed human feasibility.^11^

To date, the major efforts of most research with these dual function platforms has focused on sensor function.^6,7,10-12^ Only limited data regarding cannula durability are available, and all of which pertain to a dual needle platform.^7,8^ The impact of glucose sensing on insulin delivery and cannula survival is unclear.

The device described by Ward et al^10^ and Jacobs et al^11^ has been further refined. First successful results of the performance of a trocar version of an investigational Continuous Glucose Monitoring–Infusion Set (CGM-IS) in a clinical study with 15 type I diabetes adults were reported recently.^13^ An updated trocar-free iteration of the investigational CGM-IS was introduced by Pacific Diabetes Technologies (PDT) this year. It was our aim in this study to conduct a human trial testing the glucose sensor accuracy and infusion site patency over four days of the trocar-free CGM-IS in adults with T1D who are managed with insulin pumps and CGM.

## Methods

### Study Design

**Figure 1.**
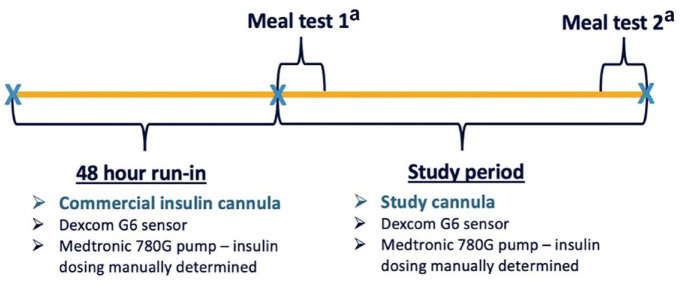
Study design overview. ^a^80 g carbohydrate standardized meal test with bolus rapid-acting insulin administered according to the participants’ established insulin to carbohydrate ratio. Meal test 1 and 2 were performed on days 1 and 4 of the study, respectively.

This was a single-center (St Vincent’s Hospital Melbourne), single-arm, non-randomized feasibility study with a two-day run-in followed by wearing a trocar-free CGM-IS (SynerG, Pacific Diabetes Technologies, Portland, Oregon) for four days. The protocol incorporated standardized meal challenges on days 1 and 4 (Figure 1). It was approved by a Human Research Ethics Committee (St Vincent’s Hospital Melbourne HREC Reference: HREC 105/19) and the trial was registered (ACTRN12619001295134).

### Participants

Insulin pump and CGM experienced adults aged 21 to 75 years with T1D duration >six months with an HbA1c between 5.8% and 10.0% (40-86 mmol/mol) willing and able to meet the protocol requirements were recruited. Pregnant woman or those contemplating pregnancy, and those with a major medical or psychiatric condition precluding their involvement in a clinical trial, were excluded.

### Study Protocol

Following consent, all participants were provided with and educated in the use of a study insulin pump (Minimed 780G, Medtronic, Northridge, California). Their established insulin delivery settings were programmed into the study pump, and insulin delivery with manual insulin dosing was commenced using a commercial insulin set for a 48-hour run-in. All participants were also provided with and educated in the use of a study blood glucose meter (Accu-Chek Guide Link, Roche, Basel, Switzerland) and a Dexcom G6 sensor (Dexcom, San Diego, California) inserted in the abdomen opposite to the investigation cannula well away from the insulin infusion site.

Following the 48-hour run-in, on study day 1, participants attended the clinical trials center at 07:00. The infusion set used during run-in was replaced with the investigational CGM-IS through which insulin delivery was commenced and the sensor initiated. Standardized test meals were eaten on the morning of days 1 and 4 (see below). All participants returned home between meal tests and delivered basal and bolus insulin according to their usual dosing regimens with the study pump through the CGM-IS. They were advised to perform at least 10 finger-stick blood glucose meter readings evenly distributed through each day.

### Glucose-sensing Cannula

**Figure 2.**
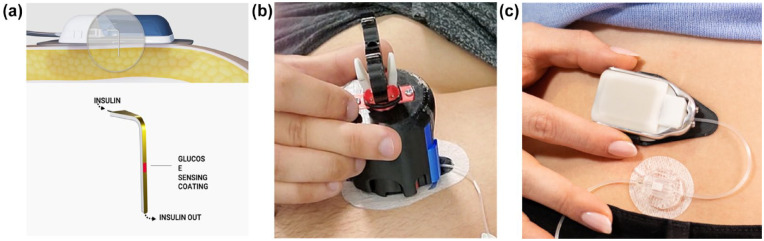
(a) Sensor element diagram, (b) sensor inserting device, and (c) photograph of sensor attached to electronic module with cannula delivering insulin.

SynerG is a second-generation trocar-free CGM-IS (Figure 2). It is an iterative development of the devices described by Ward et al^10^ and Jacobs et al.^11^ It now has a smaller foot-print planar sensing cannula design and no longer requires trocar assistance for insertion. These changes deliver a tighter insertion area with close fit, less wounded tissue, and a reduced bolus-artifact as fluid back-flow to the sensor is reduced. The outer membrane material has also been improved. In addition, the signal processing has evolved and now uses a time-sensitive sensor sensitivity-adjustment algorithm. A single blood glucose meter (BGM) value obtained during the 30-minute warm-up time was used to calibrate each individual sensor. All sensor data were processed using the same algorithm. In addition, the algorithm used can substantially remove signal artifacts corresponding to the transient signal attenuation correlated to bolus delivery.

The investigational CGM-IS is inserted subcutaneously in the anterior abdomen using a dedicated insertion device and attached to a standard insulin pump tubing with a 3-mL reservoir (Medtronic, Northridge, California). The flexible sensing cannula sensor extends to a depth beneath the skin of 6 mm at 90 degrees. The electrochemical sensing elements are located on both sides of the planar Teflon cannula with insulin delivery distal to the sensing elements. Warm-up time for the sensor is approximately 30 minutes with a single finger-stick calibration required. The cannula attaches to an Electronic Module which stores and transmits sensor current data via blue tooth to a smart phone which in turn transmits data to the cloud. The combined dimensions of the sensing cannula attached to the Electronic Module to which it is attached are 67.2 mm × 39.8 mm × 12.1 mm.

### Meal Tests

Meal tests took place in the morning following an overnight fast with the day 1 meal eaten 90 minutes following CGM-IS insertion. Arterialized blood was collected using an intravenous catheter every 10 minutes for one hour prior to a standardized 80 g carbohydrate meal and then every 15 minutes for four hours. Insulin for the meal was bolused 15 minutes prior to eating as per the participant’s established insulin to carbohydrate ratios. Samples were spun immediately and collected on ice and stored at −80°C. They were assayed for and glucose and insulin with the former duplicated on the participants’ study blood glucose meters.

### Biochemical Analyses

Plasma glucose concentrations were measured using a glucose analyzer (YSI 2300 STAT Plus Glucose Analyzer; YSI Life Sciences, Yellow Springs, Ohio). Anti-insulin antibodies and insulin concentrations were measured through an in-house radioimmunoassay (RIA) adapted from Albano et al^14^; inter-assay CVs 6.5% at 7.5 mU/L, 5.7% at 22.9 mU/L, and 5.4% at 34.2 mU/L. Plasma with anti-insulin antibodies had free insulin measured with RIA (Millipore, Billerica, Massachusetts) as previously described^15^; inter-assay CVs 3.8% at 12.6 mU/L and 3.3% at 43.3 mU/L.

### Assessments of Device Tolerance

Questionnaires were administered 15 minutes post-insertion regarding device discomfort classified as none, mild, moderate, or severe and again upon device removal at the end of day 4. Upon removal of the devices at study end, a doctor or nurse assessed the insertion site and provided a score for erythema and edema from 0 (none) to 4 (severe) according to the Draize scale.^16^

### Data Analysis

The sample size of 12 reflected resource availability and previous studies determining the feasibility of combining CGM with insulin delivery.^8^ The primary objective was to assess glucose sensor accuracy over a four-day duration as determined by mean absolute difference (MAD) for reference YSI venous blood glucose values ≤100 mg/dL and mean absolute relative difference (MARD) for reference YSI venous blood glucose values >100 mg/dL with % values within ±15 mg/dL and ±20 mg/dL at glucose concentrations <100 mg/dL and within ±15% and ±20% at ≥100 mg/dL also referred to as 15/15 and 20/20 values, respectively.^17^ Consensus error grid analyses were plotted.^18,19^ Additional analyses compared mean insulin requirements and glucose levels during run-in and while wearing SynerG at home and insulin profiles post standardized meal boluses, and unexplained glycemic excursions requiring a cannula change. Comparisons between run-in and study were made using paired Student’s *t* test and Sign rank test as appropriate.

## Results

Table 1 provides the details of the 12 participants. All completed the protocol. Profiles for individual participants summarizing bolus insulin delivery, investigational sensor glucose, YSI, meter glucose, and Dexcom data from day 1 to 4 of the study are provided (Supplementary Material 1).

**Table 1.** Baseline Characteristics of the Study Participants.

Parameter	Measure
Male/female (n)	4/8
Age (years)	47 (9)
Weight (kg)	87.6 (19.0)
T1D duration (years)	30.1 (13.2)
HbA1c (%)	7.0 (0.7)
HbA1c (mmol/mol)	53 (7)
Total daily dose insulin (units)	60.6 (25.1)

All results are expressed as mean (SD).

### Glucose Sensor Performance

**Figure 3.**
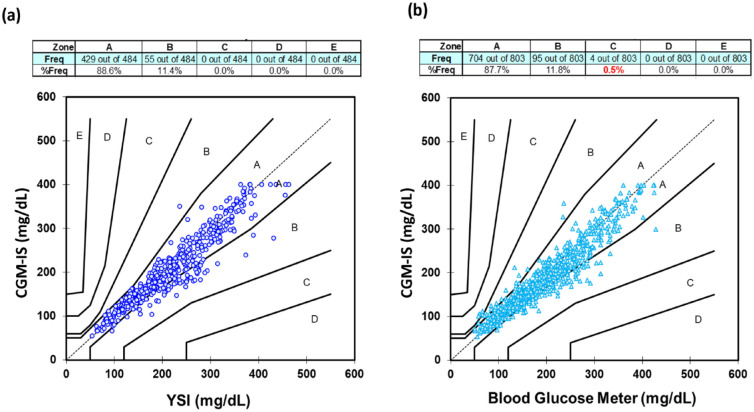
Consensus error grid for the investigational continuous glucose monitoring-infusion set (CGM-IS) with (a) YSI and (b) capillary blood glucose measurements (Accu-Chek Guide Link) as comparators.

**Figure 4.**
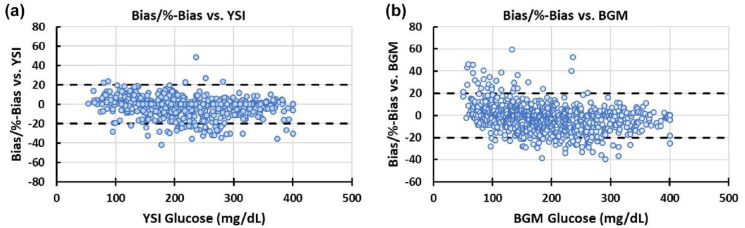
Bias/%bias plots for the continuous glucose monitoring-infusion set against (a) YSI and (b) blood glucose meter (BGM). Plots represent combined errors as bias (G − G_ref, when G_ref < cutoff = 100 mg/dL) or %-bias ([G − G_ref]/G_ref, when G_ref ≥ cutoff = 100 mg/dL).

No artifact spikes were observed. Participant 9 experienced a sensor failure during the first meal test while insulin continued to be delivered through the cannula though subsequent sensor data was obtained for the remainder of the study. Benchmarked against YSI, aggregated MARD for sensor readings during the two meal excursions for glucose was 9.0% with 484 data points. Examined separately, MARD for the day 1 and day 4 meal excursions were 8.1% and 9.8%, respectively (Table 2). The Consensus error grid plotting the CGM-IS against YSI revealed that 88.6% of values fell within zone A and 100% in zone A + B (Figure 3). Mean (SD) % bias was −3.5 (11.6) % (Figure 4). There were 35 paired YSI readings <100 mg/dL and 449 ≥100 mg/dL with 81.4% within ±15 mg/dL, ±15% and 89.9% within ±20 mg/dL, or ±20%.

**Table 2.** Individual Participant and Aggregated Mean Absolute Difference (MAD)/Mean Absolute Relative Difference (MARD) According to Meal Test, at Home, and Total.

	CGM-IS vs YSI		CGM-IS vs BGM	
Participant ID	MAD/MARD (%)	n	MAD/MARD (%)	n
001	8.3	46	9.5	69
002	12.8	45	12.1	69
003	7.8	45	11.2	55
004	8.2	46	9.4	69
005	12.3	44	10.1	69
006	8.2	43	9.3	83
007	6.2	22	6.6	51
008	7.1	43	9.4	86
009	6.5	23	7.0	42
010	10.0	45	9.1	76
011	9.5	45	9.5	75
012	8.3	37	9.2	59
**Day 1 Meal**	8.1	239	10.2	239
**At Home**	N/A	N/A	9.6	314
**Day 4 Meal**	9.8	245	8.7	250
**Total (SD)**	**9.0 (8.1)**	**484**	**9.5 (8.5)**	**803**

MAD/MARD (%): Based on the combined errors of either (G – G_ref) <100 mg/dL, or (G – G_ref)/G_ref ≥100 mg/dL cutoff. CGM-IS = Continuous Glucose Monitoring-Infusion Set. N/A = Not Applicable. YSI = Yellow Springs Instruments 2300 STAT Plus Glucose Analyzer. BGM = Blood glucose meter (Accu-Chek Guide Link, Roche). YSI data for the second test meal for Participant 007 due to a sensor failure and first test meal Participant 009 and removal of the CGM-IS at home by the participant following a line occlusion.

Bold text refers to seperate analyses on aggregated data points for the Day 1 and Day 4 meal tests, the at home period, and all (total) data points.

Benchmarked against the study blood glucose meter, MARD for the first meal excursion was 10.2% (239 data points), 9.6% (314 data points) for the at home stage, and 8.7% (250 data points) for the second meal excursion with a MARD of 9.5% (803 data points) against all blood glucose meter readings obtained during the study (Table 2). Consensus error grid with metered glucose readings as the comparator (Figure 3) revealed 87.7% of readings in zone A and 99.5% in zone A + B. Bias was −2.7 (12.4) % (Figure 4). There were 97 paired sensor and blood glucose meter readings <100 mg/dL and 706 ≥100 mg/dL with 80.1% within ±15 mg/dL, ±15% and 89.7% within ±20 mg/dL, or ±20%.

### Insulin Delivery and Glucose Levels

There were two occlusion alarms during the study. One alarm (Participant 5) occurred during the second standardized meal test, and it was elected to discontinue the bolus and give 8 of the 10.4 Unit total by subcutaneous injection with the cannula left in situ to function as a sensor till study end. The other occlusion alarm (Participant 7) occurred at home 70 hours post-insertion requiring replacement of the glucose sensing cannula with a commercially available set.

Comparing the two days of run-in using the commercially available set with the two days free living at home with the CGM-IS, mean glucose (161.3 ± 27.3 mg/dL vs 158.0 ± 25.6 mg/dL; *P* = .39), % time in range (61 ± 17% vs 64 ± 19%; *P* = .639), % time above range (35 ± 19% vs 33 ± 20%; *P* = .764), median % time below range (4 [1.5, 4.5]% vs 2.5 [1, 4]%; *P* = .408), and mean total daily insulin delivered via the pump (58.0 ± 25.4 Units vs 57.1 ± 28.8 Units; *P* = .47) did not differ.

A total of 274 insulin boluses were delivered at home and with standardized meal tests using the CGM-IS. The mean (SD) insulin bolused for day 1 and 4 test meals was 14.3 (8.2) Units and 13.0 (8.7) Units, respectively. Insulin profiles following the day 1 and 4 standardized meal tests, respectively, revealed no differences in median (interquartile range, IQR) area under the curve (9035 [7181, 122 283] vs 7861 [6536, 11 115]; *P* = .68) and peak insulin levels (56.2 [41.4, 77.3] mU/L vs 49.5 [42.0, 67.2] mU/L; *P* = .98). Time to peak insulin was greater on day 1 than day 4 (142.5 [120, 225] minutes vs 105 [90, 127.5] minutes; *P* = .018). Individual insulin profiles are provided in Supplementary Material 2. Participant 8 required large meal boluses (34.9 and 36.4 Units for day 1 and day 4 meal, respectively), which were administered as two separate boluses with the second bolus immediately following the first. It was noted that there was mild to moderate discomfort following the administration of both meal boluses and that the free insulin profile was blunted following the first bolus.

### Adverse Events and Device Tolerance

There were no episodes of major hypoglycemia, diabetic ketoacidosis (DKA), or other serious adverse events. There were no episodes with glucose levels >180 mg/dL in conjunction with ketones >0.6 mmol/L. Nine participants reported no discomfort and three reported mild transient discomfort at 15 minutes post-insertion. At other time points, no discomfort was recorded. On removal of the device, the Draize scale assessment revealed no erythema in nine participants, very slight barely noticeable erythema in two, and well-defined erythema in one. Seven participants had no edema and five had very slight edema.

## Discussion

The first commercially available sensor-augmented insulin pump (Minimed Paradigm 512/712) became available in 2003. In the intervening 20 years, we have continued to rely on separate insertions for glucose sensing and insulin delivery as there are still no commercially available platforms combining the two functions. Most CGM manufacturers advise that their sensor be placed at least 2.5 and 3.0 cm away from the insulin infusion site.^20-22^ To the best of our knowledge, this is the first in human free-living study of a trocar-free CGM-IS evaluating both glucose sensing and insulin delivery functions. Our data support feasibility of the system as it was easy to insert, well tolerated over wear time, and neither glucose sensing nor insulin delivery appeared compromised into the fourth day of wear.

The investigational CGM-IS employed a glucose oxidase–based electrochemical sensor. Although other approaches may have different susceptibilities to interference, electrochemical sensing using glucose oxidase remains the most robust option. For example, while optical fluorescence sensing has potential advantages including less interference by phenols, greater sensitivity at low glucose levels, and less requirement to recalibrate in response to peri-sensor tissue reactions, sensor accuracy is lower than with electrochemistry.^23,24^ Microdialysis and microperfusion catheters sample subcutaneous interstitial fluid by a semipermeable hollow fiber with glucose analyzed by a sensing electrode located externally which makes these devices less prone to biofouling. Although valuable as research tools, they are expensive, bulky, and complex, posing challenges in implementation in an ambulatory setting precluding them from day-to-day use.^25^

While earlier iterations, which operated at +600 mV, of the device evaluated in our study revealed interference by insulin preservatives,^10^ this was overcome by modifying the sensor chemistry for operation at +175 mV which eliminated the phenolic artifact. Our studies with the investigational CGM-IS confirmed no evidence of interference.

Nor was there evidence of a clinically significant impact on sensor values by local dilution or increased glucose uptake at the depot site as evidenced by sensor values benchmarked against YSI and the study glucose meter.^10,11^ Also, while a formal comparison was not performed, the Dexcom sensor placed well away from the infusion site provided 15/15 and 20/20 values against YSI of 76.5% and 86.0% with a bias of −2.4% compared with 81.4% and 89.9% with a bias of −3.5% for the CGM-IS. The G6 accuracy metrics reported here should be viewed in context with those from a previous Dexcom-sponsored study where the 15/15, 20/20, and MARD values were 83.3%, 93.9%, and 9.0%, respectively.^26^

The integrity of insulin delivery is at least equally as important as the accuracy of glucose measurements. Mean glucose and average daily insulin dosing during the two free living days at home benchmarked against the 48-hour run-in revealed no significant differences. The two days with the test meals were excluded from the comparison with run-in, as the carbohydrate intake, physical activity, psychological stress, and insulin dosing would not have reflected an average day. Our data suggest that insulin delivery did not appear to be compromised over the four days as free insulin excursions in the blood following the bolus for the test meals on days 1 and 4 of the study were similar. However, the participant numbers were small and definitive larger, longer duration studies are required.

Despite being a prototype, the CGM-IS device was well tolerated with a minority of participants reporting mild transient discomfort. There were no significant tissue reactions observed over the four days of wear. In its present form, the device is relatively bulky, and we would expect the form factor to be refined and the size of the device reduced with further development.

Limitations include the short duration and the small number of data points in the hypoglycemic range. Therefore, analysis of the data against the iCGM evaluation criteria was not performed as a sufficient dataset was not available in several categories. Future studies aim to explore sensor performance at low glucose levels in greater detail with the insulin delivery and glucose sensing over seven days of wear. We also acknowledge that two investigational devices worn simultaneously with and without insulin delivery would have provided more robust data regarding the impact of insulin on sensor function. However, the burden imposed by the protocol was already significant. The study data will be used to refine the processing algorithm with the aim to generate a glucose measurement in real time.

Strengths of the study included the two standardized meals providing important data on glucose sensing and insulin delivery under clinically relevant conditions as did the at home period between the two meal tests. Finally, while we recognize that the CGM-IS is a prototype, the device tolerance data is also of relevance.

Combining insulin delivery and glucose sensing has significant clinical implications given the potential to reduce the physical, psychological, and intellectual burden imposed on people using AID systems. In addition, the colocation of these two functions could enhance communication between AID components. Finally, a combined subcutaneous platform may have health economic and environmental implications in terms of resources used for manufacturing and packaging. Our human feasibility study represents an early but important step, and further research is needed to ensure that the device is durable over an extended period without discrepancies between sensor and cannula survival.

## Conclusions

This report details the first feasibility study in humans of a CGM-IS used at home. No interference between the glucose sensing and insulin delivery functions was apparent and further development is warranted. Ultimately, it is expected that combining glucose sensing and insulin delivery in a single insertion device may reduce the burden of care and ultimately improve the lives of people living with diabetes.

## Supplemental Material

sj-pptx-1-dst-10.1177_19322968241236771 – Supplemental material for Combining an Electrochemical Continuous Glucose Sensor With an Insulin Delivery Cannula: A Feasibility Study Supplemental material, sj-pptx-1-dst-10.1177_19322968241236771 for Combining an Electrochemical Continuous Glucose Sensor With an Insulin Delivery Cannula: A Feasibility Study by Cheng Yi Yuan, Bella Halim, Yee W. Kong, Jean Lu, Ralph Dutt-Ballerstadt, Peter Eckenberg, Ken Hillen, Anh Koski, Vlad Milenkowic, Emma Netzer, Varuni Obeyesekere, Solomon Reid, Catriona Sims, Sara Vogrin, Huan-Ping Wu, Thomas Seidl and David N. O’Neal in Journal of Diabetes Science and Technology

sj-pptx-2-dst-10.1177_19322968241236771 – Supplemental material for Combining an Electrochemical Continuous Glucose Sensor With an Insulin Delivery Cannula: A Feasibility Study Supplemental material, sj-pptx-2-dst-10.1177_19322968241236771 for Combining an Electrochemical Continuous Glucose Sensor With an Insulin Delivery Cannula: A Feasibility Study by Cheng Yi Yuan, Bella Halim, Yee W. Kong, Jean Lu, Ralph Dutt-Ballerstadt, Peter Eckenberg, Ken Hillen, Anh Koski, Vlad Milenkowic, Emma Netzer, Varuni Obeyesekere, Solomon Reid, Catriona Sims, Sara Vogrin, Huan-Ping Wu, Thomas Seidl and David N. O’Neal in Journal of Diabetes Science and Technology
