# Supplementary material for: Combining an Electrochemical Continuous Glucose Sensor With an Insulin Delivery Cannula: A Feasibility Study
Source: J Diabetes Sci Technol. 2024 Mar 16;18(6):1273–80. doi: 10.1177/19322968241236771 (PMC11535351; doi:10.1177/19322968241236771)
Supplement: sj-pptx-1-dst-10.1177_19322968241236771 – Supplemental material for Combining an Electrochemical Continuous Glucose Sensor With an Insulin Delivery Cannula: A Feasibility Study [file sj-pptx-1-dst-10.1177_19322968241236771.pptx]

## Slide 1
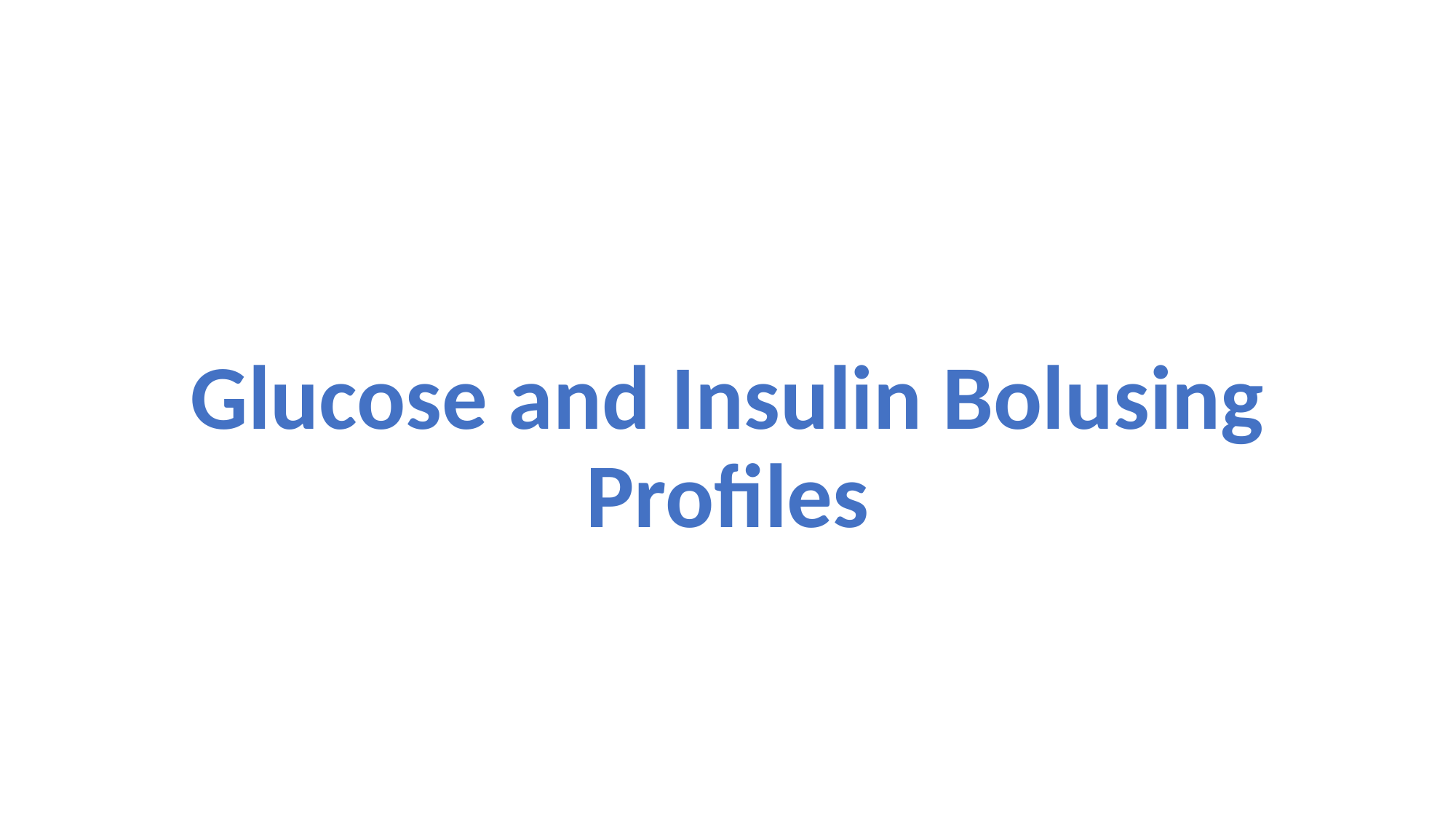

Glucose and Insulin Bolusing Profiles

## Slide 2
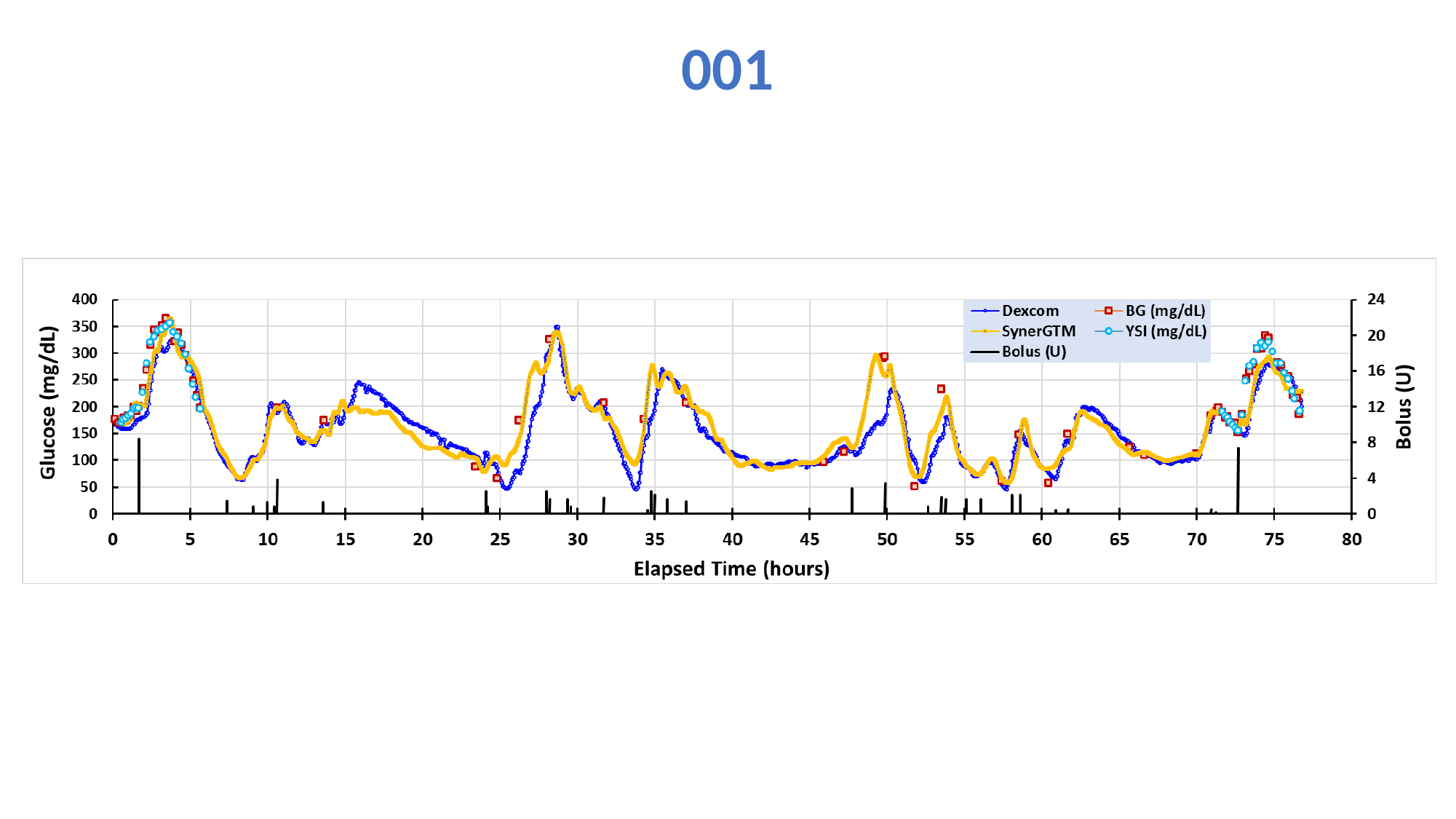

# 001

## Slide 3
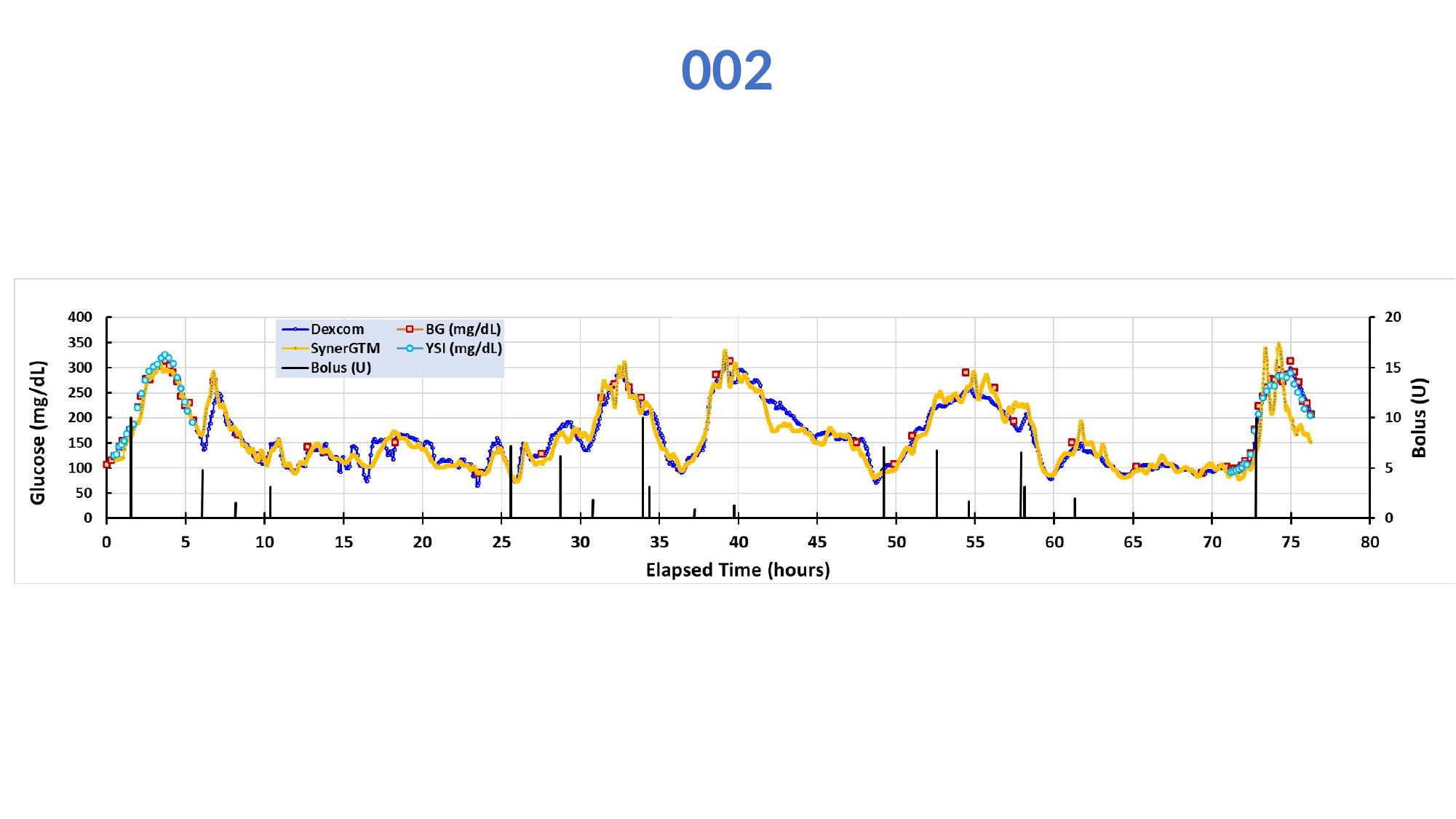

# 002

## Slide 4
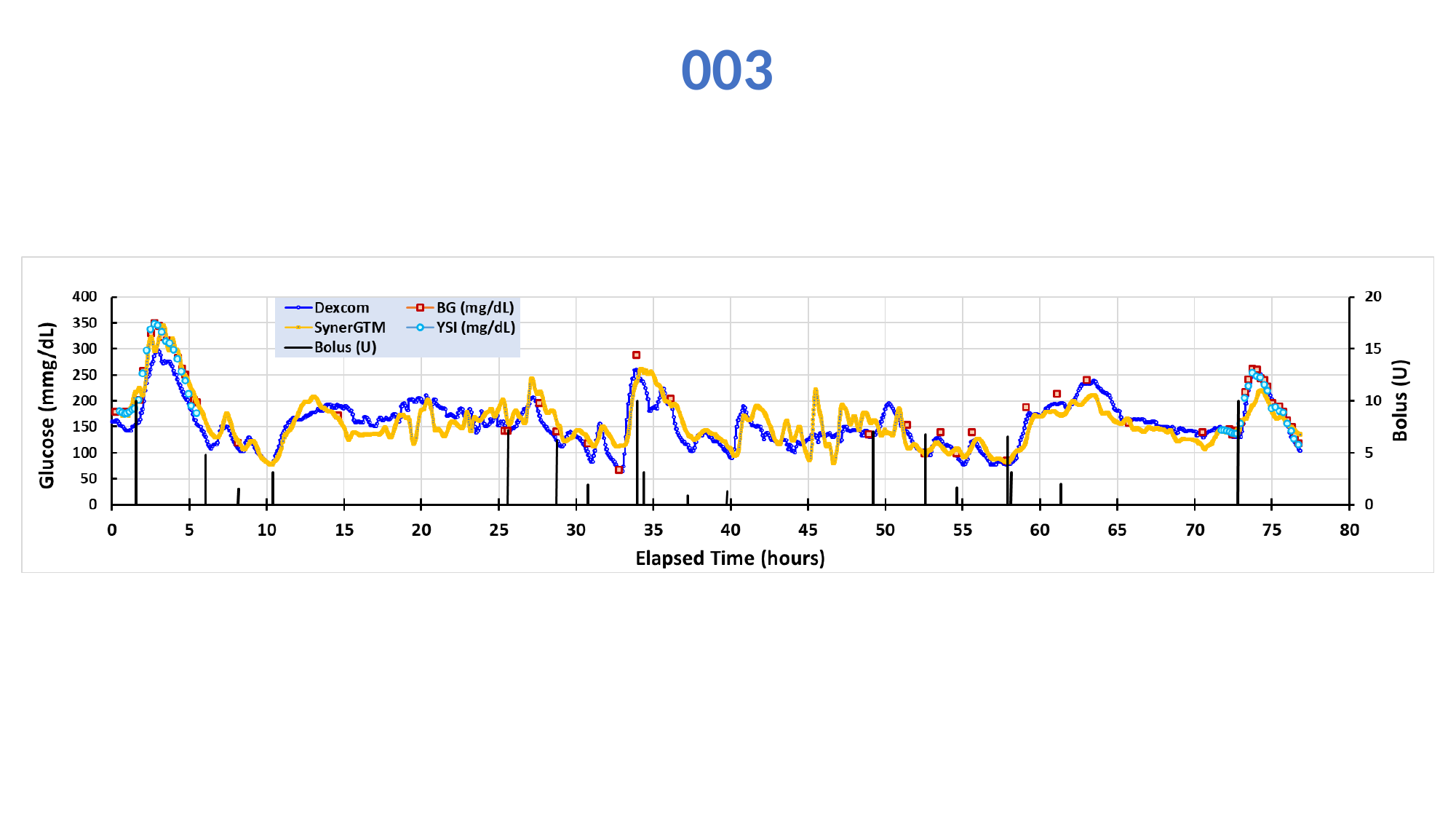

# 003

## Slide 5
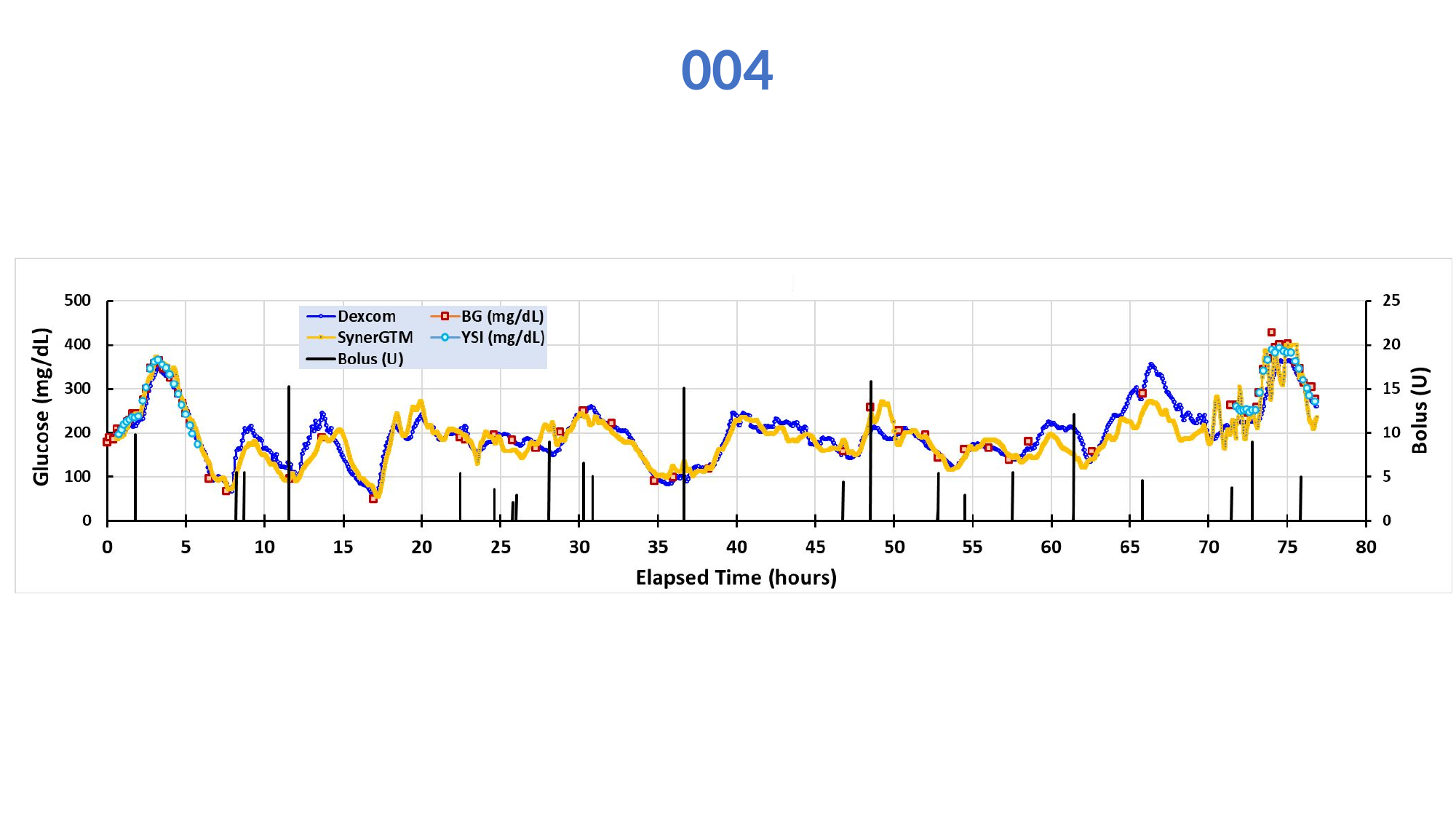

# 004

## Slide 6
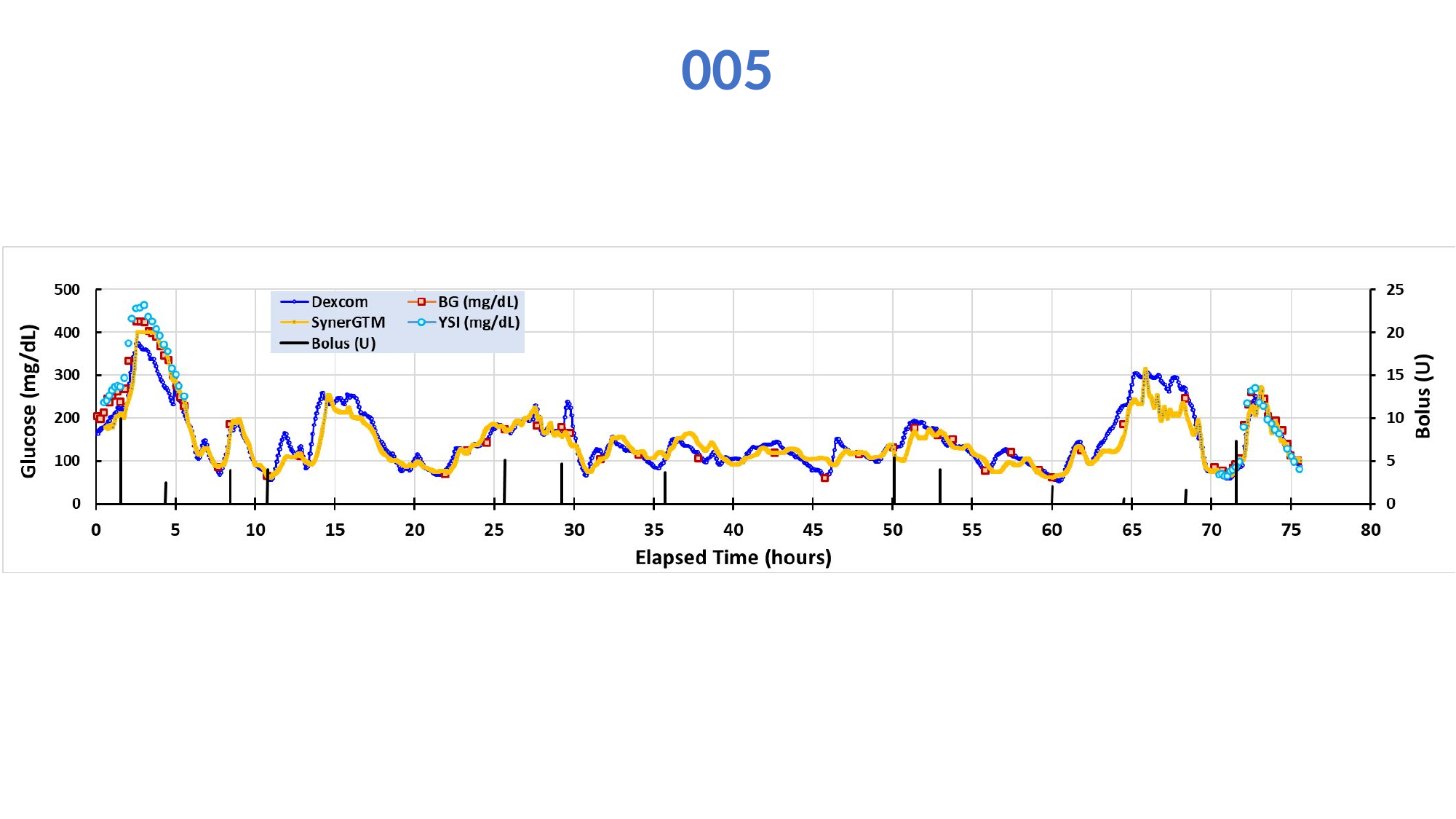

# 005

## Slide 7
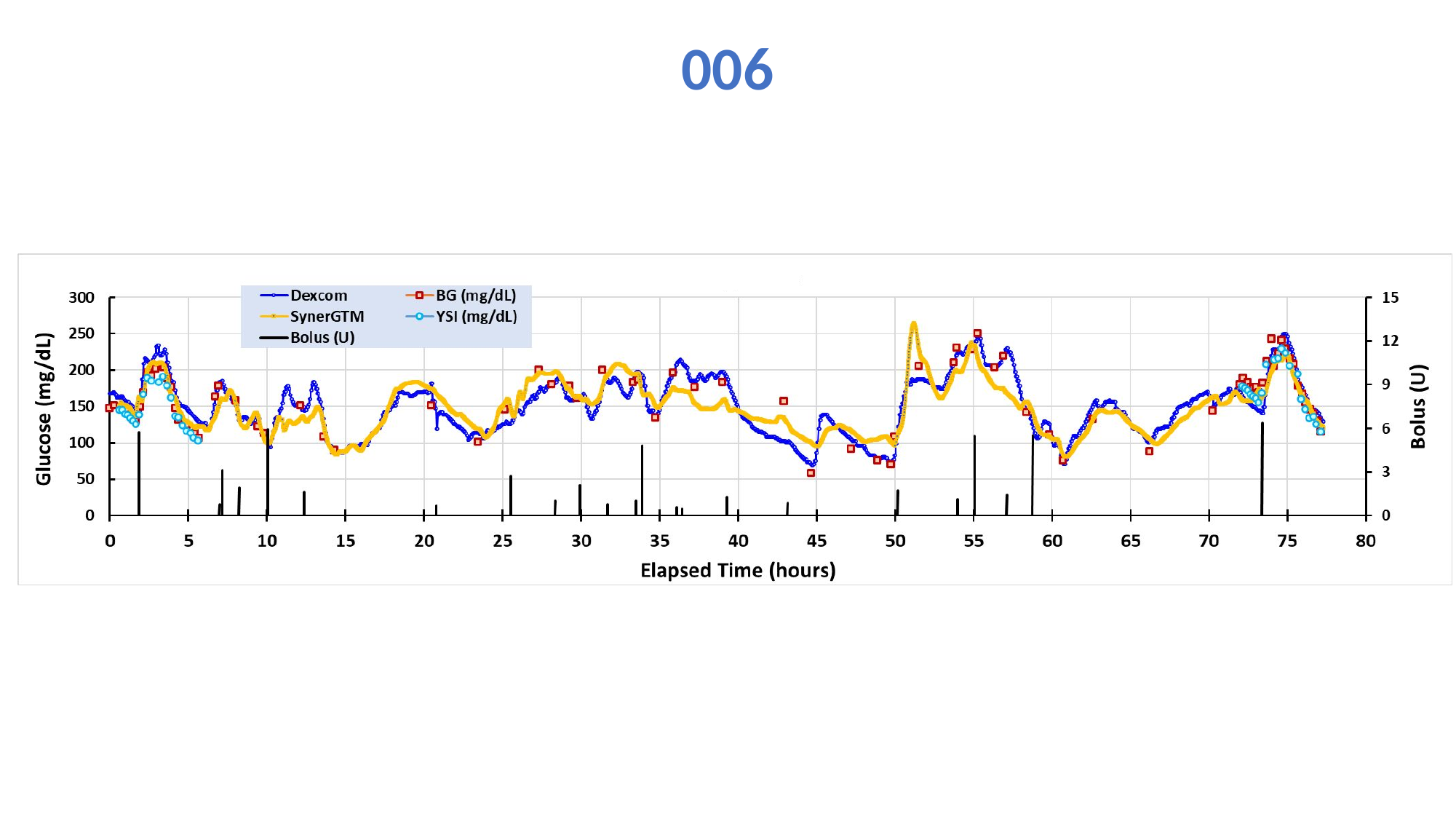

# 006

## Slide 8
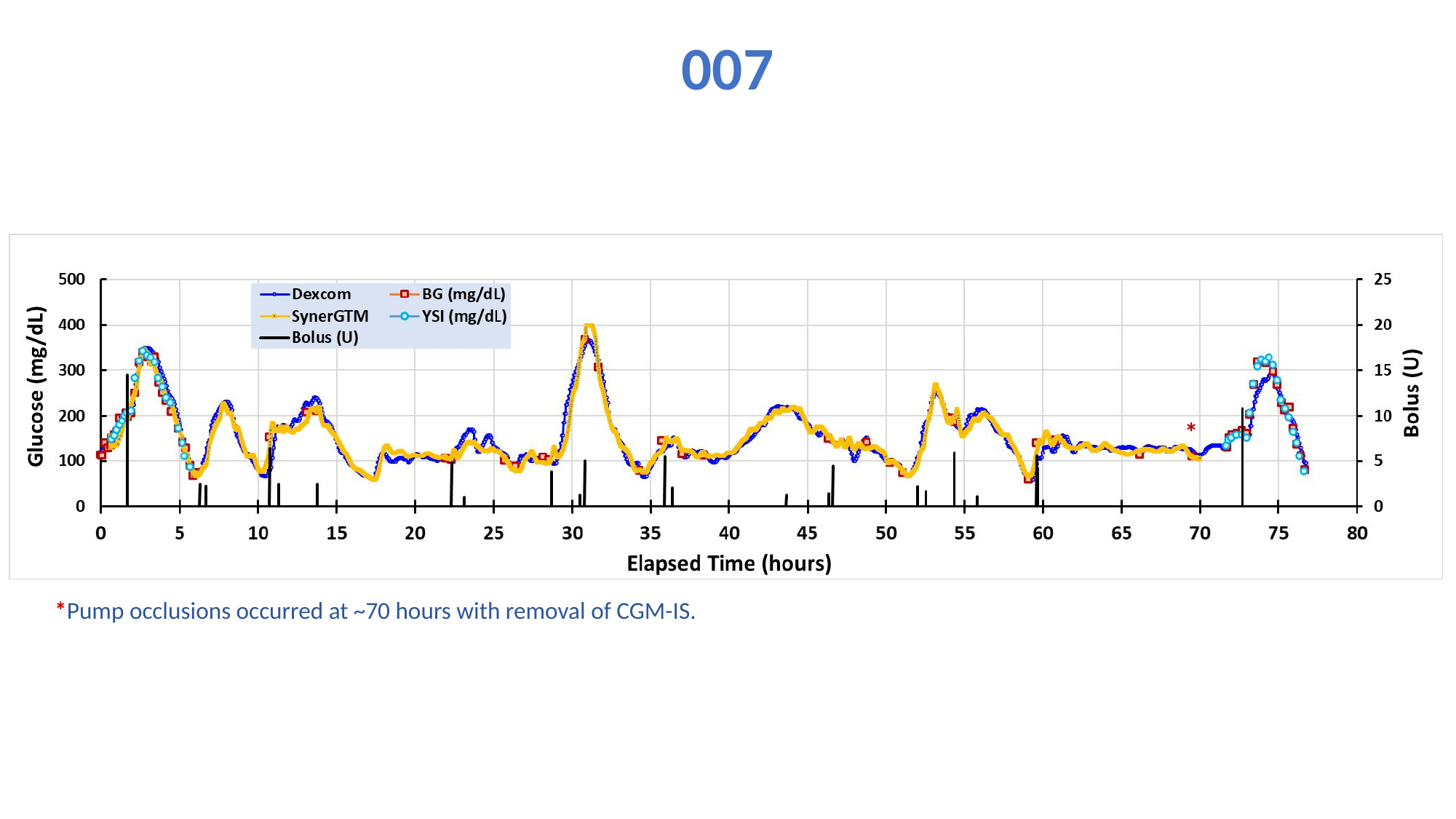

# 007
*
*Pump occlusions occurred at ~70 hours with removal of CGM-IS.

## Slide 9
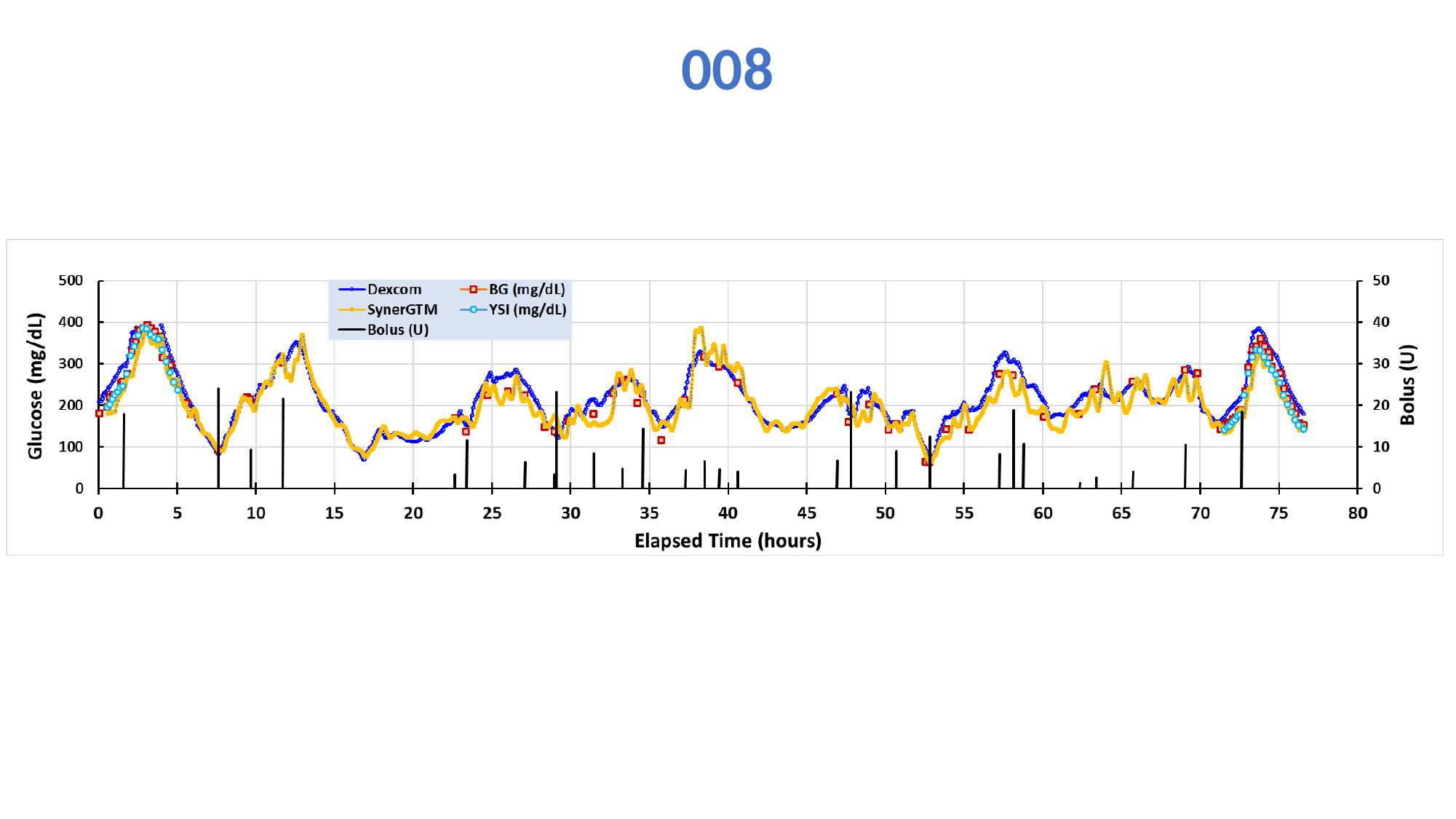

# 008

## Slide 10
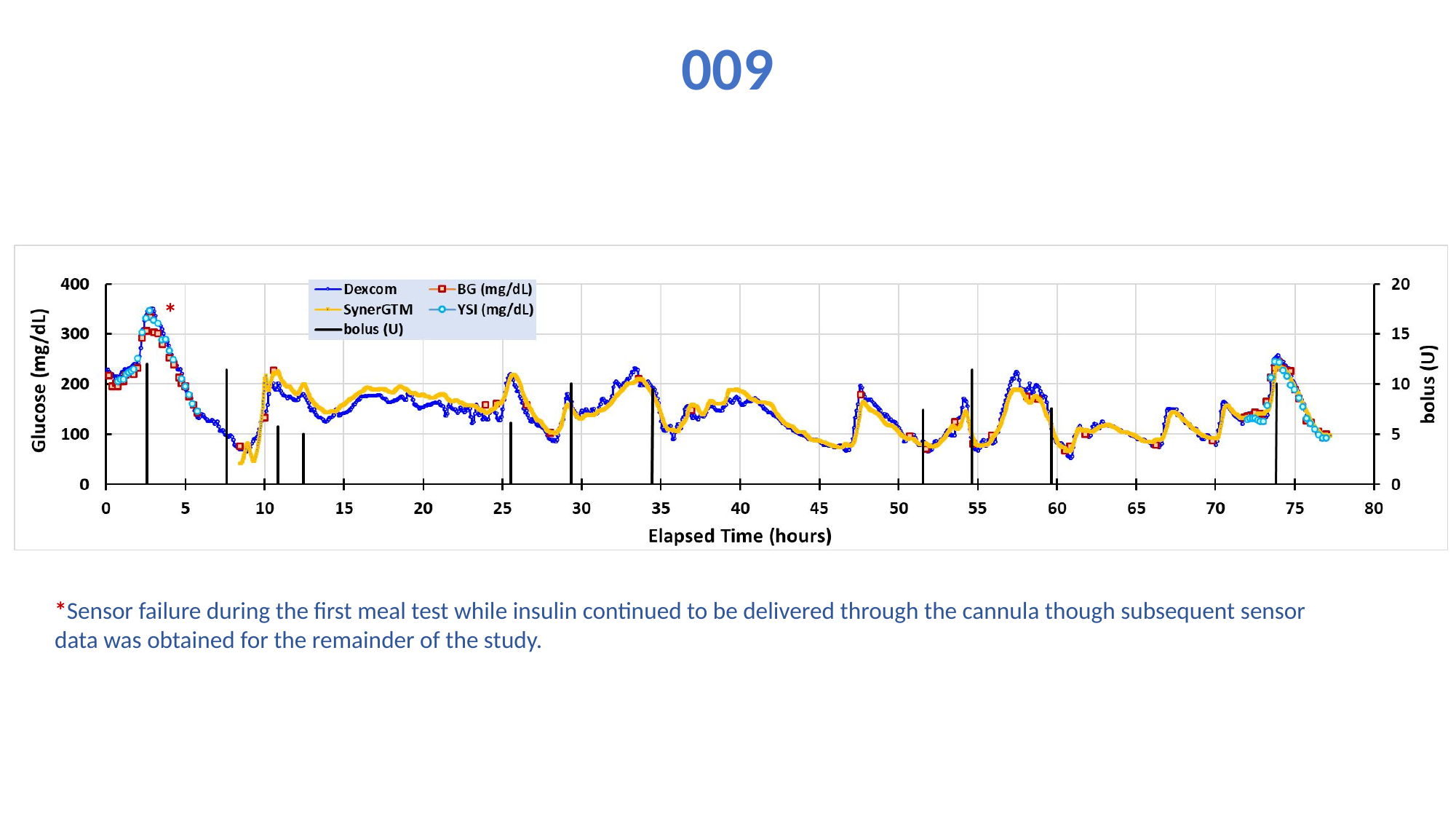

# 009
*
*Sensor failure during the first meal test while insulin continued to be delivered through the cannula though subsequent sensor data was obtained for the remainder of the study.

## Slide 11
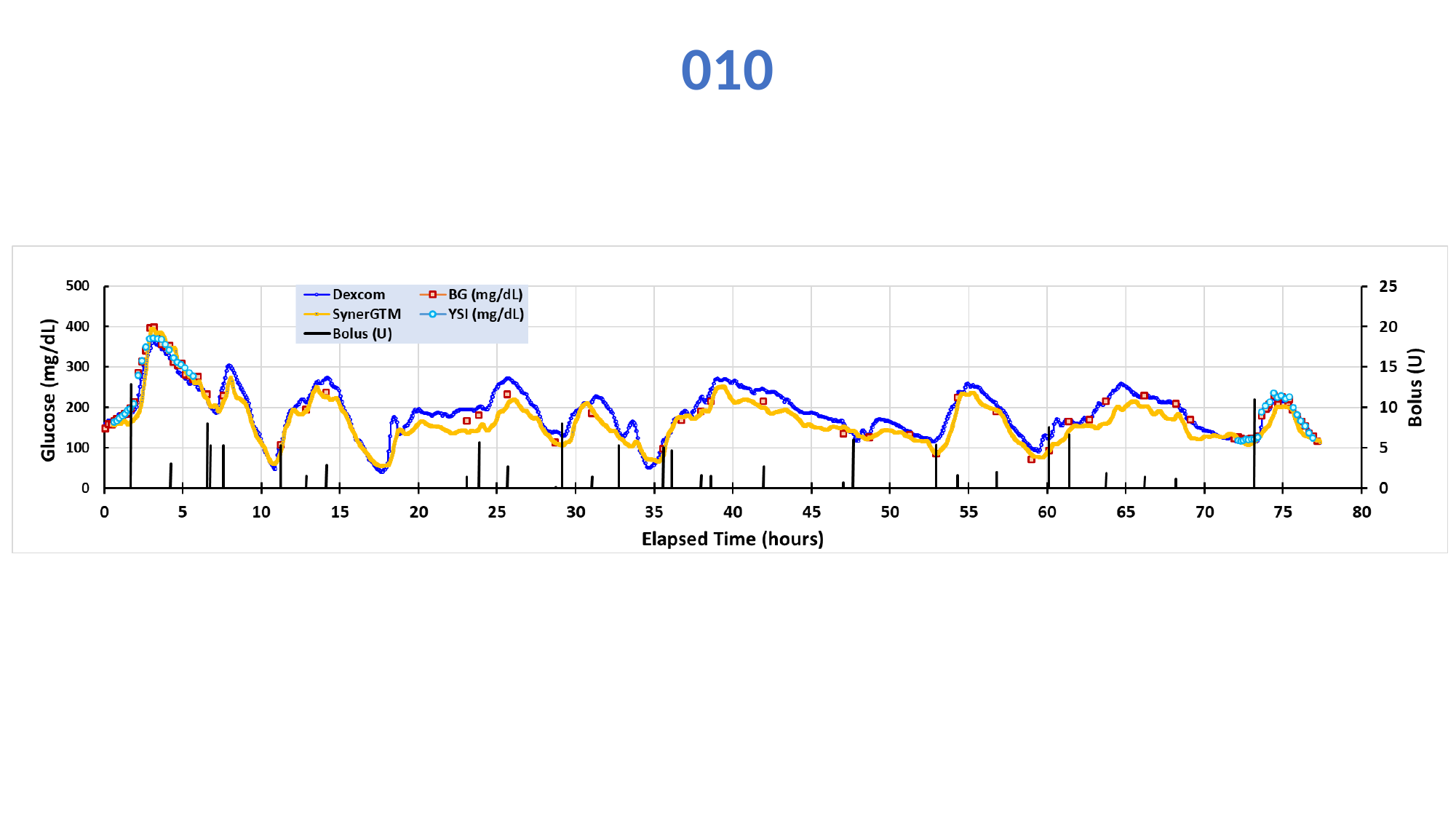

# 010

## Slide 12
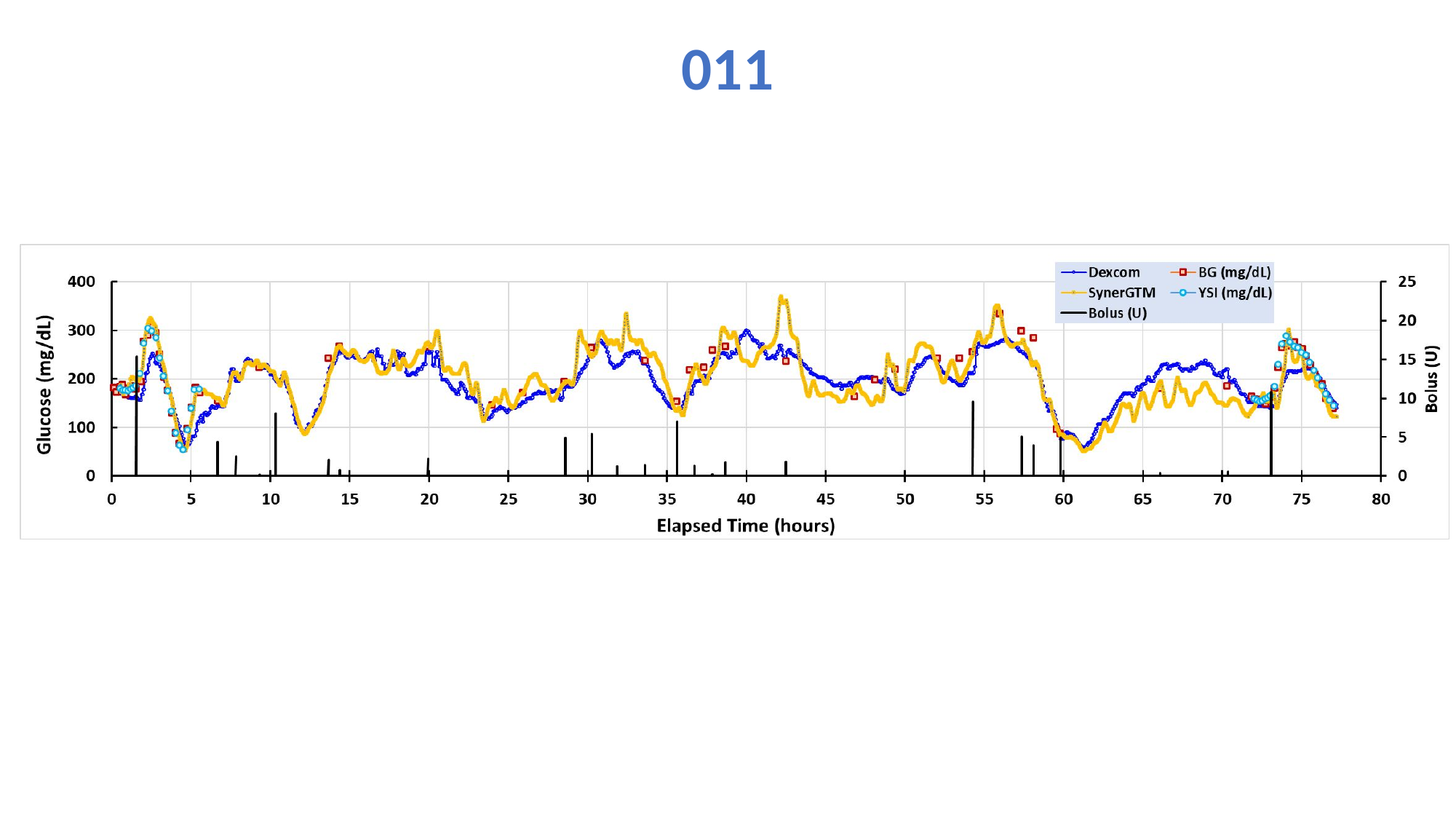

# 011

## Slide 13
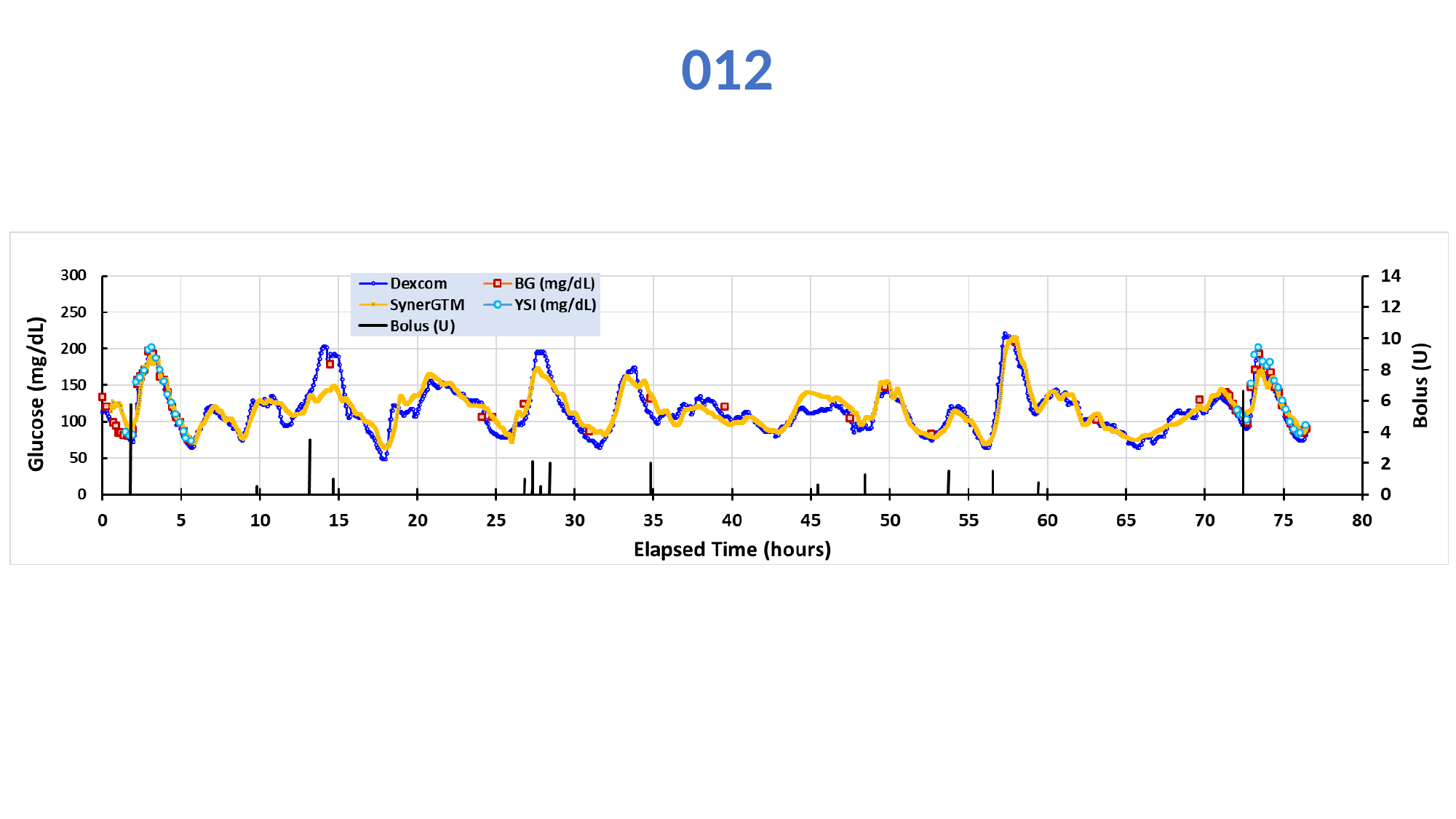

# 012
