# Supplementary figures and images for: Combining an Electrochemical Continuous Glucose Sensor With an Insulin Delivery Cannula: A Feasibility Study
Source: J Diabetes Sci Technol. 2024 Mar 16;18(6):1273–80. doi: 10.1177/19322968241236771 (PMC11535351; doi:10.1177/19322968241236771)

## Slide 1
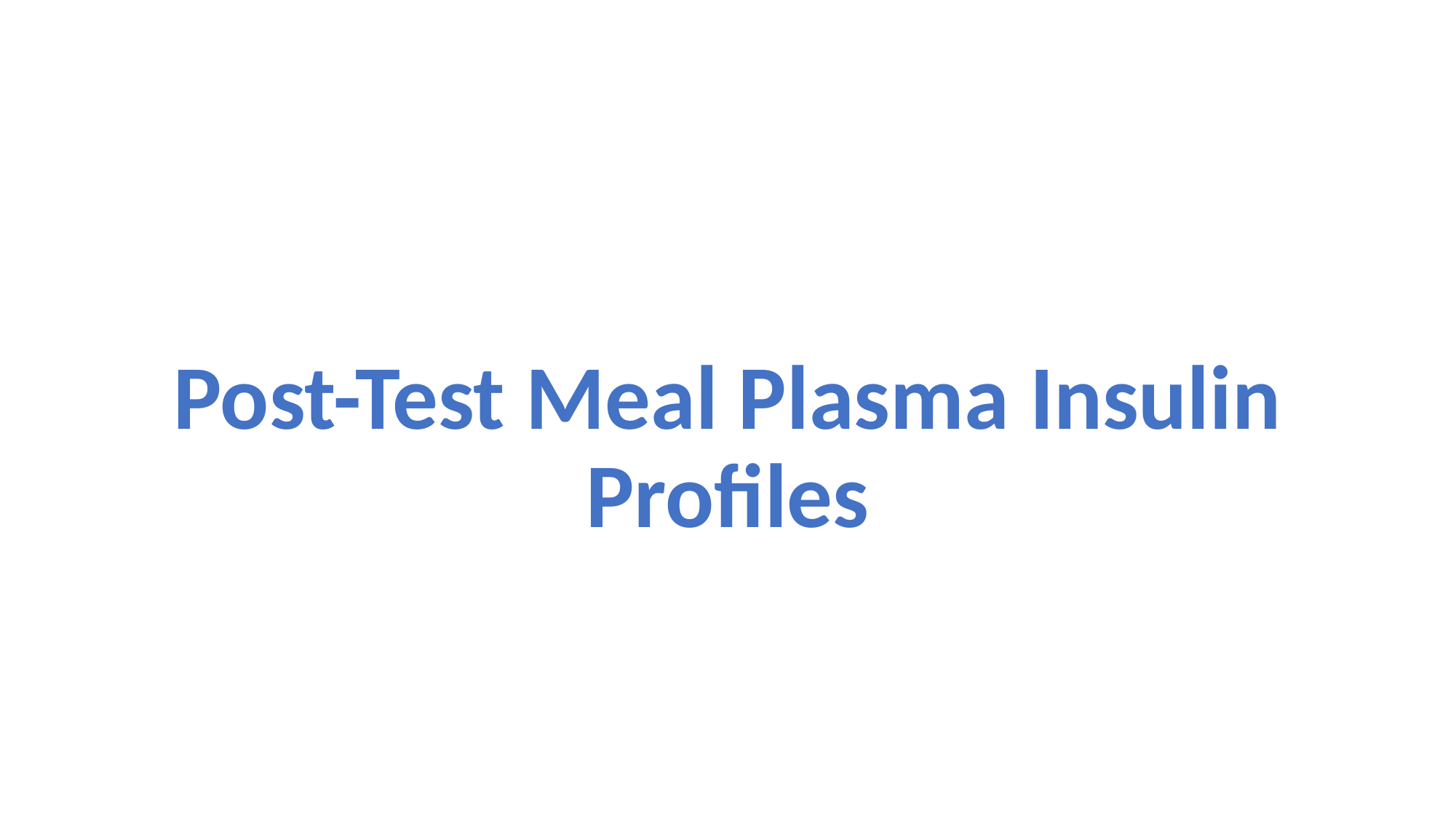

Post-Test Meal Plasma Insulin Profiles

## Slide 2
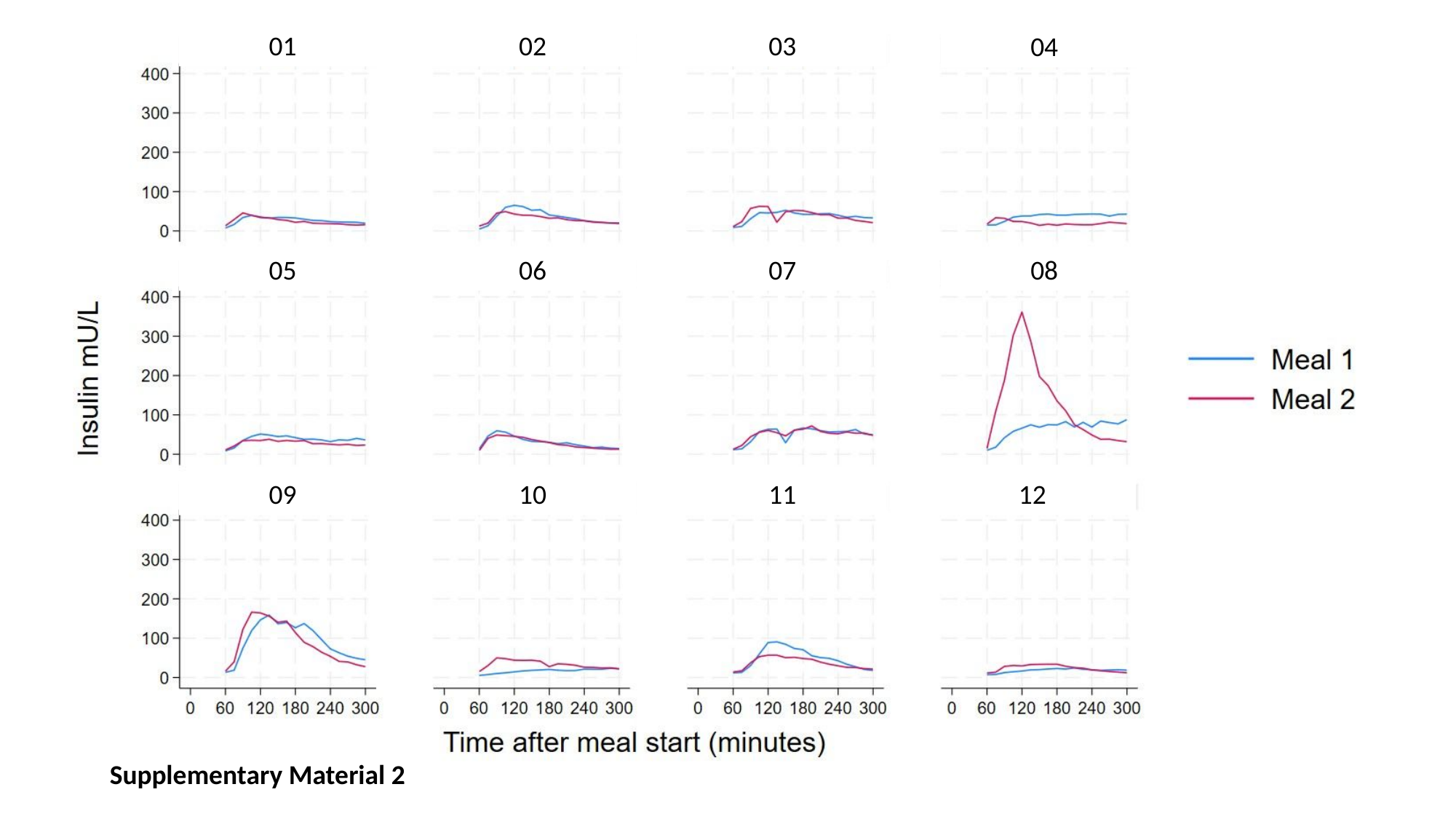

01
02
03
04
05
06
07
08
09
10
11
12
Supplementary Material 2

Supplement: sj-pptx-2-dst-10.1177_19322968241236771 – Supplemental material for Combining an Electrochemical Continuous Glucose Sensor With an Insulin Delivery Cannula: A Feasibility Study [file sj-pptx-2-dst-10.1177_19322968241236771.pptx]
